# Metal Contamination and the Epidemic of Congenital Birth Defects in Iraqi Cities

**DOI:** 10.1007/s00128-012-0817-2

**Published:** 2012-09-16

**Authors:** M. Al-Sabbak, S. Sadik Ali, O. Savabi, G. Savabi, S. Dastgiri, M. Savabieasfahani

**Affiliations:** 1 Department of Obstetrics and Gynecology, Al Basrah Maternity Hospital, Al Basrah Medical School, P.O. Box 1633, Basrah, Iraq; 2 Department of Prosthodontics, School of Dentistry, Isfahan University of Medical Sciences, Isfahan, Iran; 3 National Public Health Management Center, School of Medicine, Tabriz University of Medical Sciences, Tabriz, Iran; 4 School of Public Health, University of Michigan, 1415 Washington Heights, EHS Room Number M6016, Ann Arbor, MI 48109-2029 USA

**Keywords:** Iraq, Metal exposure, Human birth defects, Folate-dependent birth defects

## Abstract

Between October 1994 and October 1995, the number of birth defects per 1,000 live births in Al Basrah Maternity Hospital was 1.37. In 2003, the number of birth defects in Al Basrah Maternity Hospital was 23 per 1,000 live births. Within less than a decade, the occurrence of congenital birth defects increased by an astonishing 17-fold in the same hospital. A yearly account of the occurrence and types of birth defects, between 2003 and 2011, in Al Basrah Maternity Hospital, was reported. Metal levels in hair, toenail, and tooth samples of residents of Al Basrah were also provided. The enamel portion of the deciduous tooth from a child with birth defects from Al Basrah (4.19 μg/g) had nearly three times higher lead than the whole teeth of children living in unimpacted areas. Lead was 1.4 times higher in the tooth enamel of parents of children with birth defects (2,497 ± 1,400 μg/g, mean ± SD) compared to parents of normal children (1,826 ± 1,819 μg/g). Our data suggested that birth defects in the Iraqi cities of Al Basrah (in the south of Iraq) and Fallujah (in central Iraq) are mainly folate-dependent. This knowledge offers possible treatment options and remediation plans for at-risk Iraqi populations.

It is old knowledge that exposure to chemicals can harm human reproduction. Ancient Romans were aware that lead (Pb) poisoning can cause miscarriage and infertility (Gilfillan 1965; Retief and Cilliers 2006). Today it is well established that human pregnancy and fetal development are susceptible to parents’ environmental exposure to chemical, biological and physical agents (Mattison 2010). A new concept of the developmental origins of health and disease has also emerged which is defined as the process through which the prenatal environment, or the environment during infancy, shapes the long-term control of tissue physiology and homeostasis (Barker 2004). We know that even slight perturbations caused by chemical exposures during sensitive periods of fetal development can lead to increased risks of disease throughout the life of an individual (Sutton et al. 2010).

Pregnant mothers and their growing fetuses are especially vulnerable to exposure to pollutants. Air and water pollution, exposure to toxic metals, and exposure to persistent and volatile organics have been linked to adverse pregnancy and developmental outcomes (Landrigan et al. 2004; Bocskay et al. 2005). Recently, an unusual number of birth defects, in many bombarded Iraqi cities, has raised international concern and a few relevant studies have been published (Alaani et al. 2011a; 2011b; Al-Ani et al. 2010). Following bombardment, severe contamination of water, soil, and air can occur. Metal contamination of the public after bombardment has been reported (Jergovic et al. 2010). Jergovic et al. (2010) examined the blood serum metal content of the Croatian population in areas with “moderate fighting” versus “heavy fighting”. They found significantly higher levels of metals in populations from areas with heavy fighting. Those areas had been targeted for repeated bombardments by the North Atlantic Treaty Organization in 1991 and 1995. Various metals are contained in US small arms ammunition, and are contained in US bombs (Departments of the Army, the Navy, the Air Force, Joint Technical Bulletin, 1998; US Department of the Army Technical Manual, 1990).

Intermittent bombing of populated cities in Iraq has occurred since 1991. Most significant was the bombardment of Fallujah, a city in central Iraq, and Al Basrah, a city in southern Iraq. Fallujah was heavily bombed in 2004. Subsequently, unusual numbers of birth defects have been surfacing in that city. Al Basrah was also a target of heavy bombing (December 1998, March and April 2003). Similar to Fallujah, after the 2003 invasion and occupation of Iraq, the medical staff in Al Basrah Maternity Hospital has been witnessing a pattern of increase in congenital birth defects. Based on these observations, many suspect that pollution created by the bombardment of Iraqi cities has caused the current birth defect crisis in that country (Al-Hadithi et al. 2012).

In the present article, we have reported on 56 Fallujah families’ hair metal levels and the kinds of birth defects documented in these families. The Fallujah study was conducted in 2010. We have also presented a year-to-year account of the types and numbers of birth defects in Al Basrah Maternity Hospital from 2003 to 2011. Our aim was to examine the populations of Fallujah and Al Basrah for possible metal exposure. In our samples from Al Basrah, we were looking at three tissues (hair, toenail, and teeth) and wanted to determine which tissue provides a better medium for metal analysis.

## Materials and Methods

**Fig. 1:**
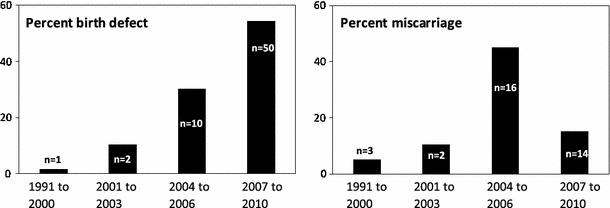
Percentage of birth defects and percentage of miscarriages among 56 Fallujah families who had come to Fallujah General Hospital for treatment or delivery between May and August 2010

Between May and August 2010, 56 Fallujah families were recruited into an epidemiological “case study” project. Cases (n = 46) and controls (n = 10) had come to Fallujah General Hospital for delivery or treatment. Using a questionnaire, information on reproductive history of families and the parents’ siblings, residence history, health and disease during pregnancy, drug use during pregnancy, smoking and alcohol use, source of water for the family, and exposure to potential war contaminants was collected. Hair samples were collected from all members of the family (mothers, fathers and the children) and patient consent was obtained at the same time. Participants had lived continuously in Fallujah since 1991. Percentages of birth defects and miscarriages were determined (Fig. 1); grouping of the years was based on “before” and “after” the 2003 attacks. For the period of 2003 onward, we used 3-year intervals. The remaining single-year data, from 2010, was added to the last group. For each of the groups, the percentages of birth defects and miscarriages were determined by totaling live births and multiplying that figure by 100, then dividing the value by the total number of birth defects or miscarriages, respectively. The metal content of hair samples was determined by inductively coupled plasma mass spectrometry (ICPMS). We collected hair rather than blood samples, since it is difficult to obtain and transport blood samples in a war zone.

In Al Basrah, records of the Department of Obstetrics and Gynecology at Al Basrah Maternity Hospital were examined for numbers and types of diagnosed and reported birth defects during 1994 and then from 2003 to 2011 (Table 1). Birth defects in the newborns were diagnosed by certified medical doctors. Additionally, between September 2011 and January 2012, twenty-eight families who had come to Al Basrah Maternity Hospital for treatment or delivery were recruited into this study by local physicians and patient consent was obtained. Hair and toenail samples were collected from two groups: parents who recently had a child with a birth defect (n = 14); and parents who had a normal child (n = 14). Only 6 samples of hair and 6 samples of toenails from parents of children with defects were sufficient in weight to be analyzed for metal levels.

**Table 1:** Yearly account of birth defects per 1,000 live births in Al Basrah Maternity Hospital from 2003 to 2011

Year	Central Nervous System Defects			Other defects				
	Hydrocephalus	Anencephaly	Spina Bifida	Limb deformity	Omphalocele	Short extremities	Multiple birth defects	Total
2003	3	5	4	3	2	2	4	23
2004	3	6	8	3	4	3	8	34
2005	3	4	5	5	4	4	9	34
2006	6	8	8	3	5	2	12	44
2007	6	8	6	3	4	3	15	45
2008	2	8	4	1	3	2	15	35
2009	6	8	7	4	4	4	15	48
2010	5	4	3	3	2	2	10	29
2011	4	6	6	4	2	1	14	37

Tooth samples were simultaneously collected at the Al Basrah Dental School from parents of normal children (n = 10) and parents of children with cardiac and neural tube defects (n = 12). Only 5 of each group had sufficient and suitable tooth tissue for metal analysis. Parents did not smoke or drink. Two samples of deciduous teeth were collected from children with birth deformity who had survived. Deciduous teeth from normal children were provided by the School of Dentistry at Isfahan University of Medical Sciences (n = 18, two samples were analyzed). Patient/parental consent was obtained.

Hair samples’ treatment, digestion and their analysis for Fallujah samples followed Batista et al. 2009 without modification. XSERIES 2 ICP-MS (Thermo Fisher Scientific, Germany) was used in the standard configuration, with ASX-510 auto-sampler (Cetac, USA). Instrument optimization was by auto-tune function, when required. The instrument parameters were: RF Power (W) 1400, Cool Gas Flow (L/min) 13, Auxiliary Gas Flow (L/min) 0.8, Nebuliser Gas Flow (L/min) 0.85–0.90, Sample Uptake Rate (mL/min) 0.4 approx., Sample Introduction System Concentric nebuliser with low-volume impact bead spray chamber (not cooled) and one-piece torch (1.5 mm ID injector); Cones Nickel, Xi Design; Detector Simultaneous pulse/analogue; Uptake Time 25 s at 50 rpm; Stabilization Delay 10 s at 17 rpm; Wash Time 40 s at 50 rpm, Survey Runs 1—scanning; Main Runs 3—peak jumping; Number of Points per Peak 1; Dwell Time/Point 5—50 ms; Number of Sweeps/Replicate 25. Internal Standardization Technique Interpolation, using 6Li, 45Sc, 115In, 159 Tb. Total Time per Sample 2:45 min.

Toenail and hair samples from Al Basrah were prepared and analyzed as follows: All reagents used were analytical grade or better. All aqueous solutions were prepared using deionised water (18.2 MΩ Millipore, UK). A multi-element standard and single element standard for the internal standards (SPEX CertiPrep, UK) were used as calibration standard and internal standard respectively for ICP-MS analysis. Concentrated nitric acid (HNO3) and 30% v/v hydrogen peroxide (H2O2), (BDH Aristar,UK) were used for the dissolution of samples. Analyses of toenail and hair samples were conducted at the Inorganic Geochemistry laboratories of the British Geological Survey (Nottingham, UK). Toenail and hair samples were washed thoroughly following a slightly modified version of the protocol described by Button et al. (2009), which is comparable to several published methods (Slotnick et al. 2007). Visible exogenous material was firstly removed using plastic forceps and a clean quartz fragment. Samples were then placed in clean glass vials and sonicated for 5 min using 3 mL of acetone, rinsed first with 2 mL of deionised water then 2 mL of acetone, sonicated for 10 min in 3 mL of deionised water then twice rinsed with 3 mL of deionised water, ensuring complete submersion of the sample during each step. The final rinse solution (3 mL) was retained for immediate analysis by ICP-MS to ensure removal of exogenous contamination was complete. The supernatants from each step of the washing procedure were combined and reduced to dryness in PFA vials (Savillex, USA) on a graphite hot block at 80°C. The residue was then reconstituted in 3 mL of 1% HNO3 for analysis by ICP-MS. After washing, toenails were left to dry at room temperature in a clean laminar flow hood. Certified reference materials GBW 07601 human hair and^1^ and NCS ZC 81002b human hair (NCS Beijing, China) were used throughout.

Both the digestion and analytical method follow the procedure described in Button et al. (2009). Toenail samples were acid digested for total elemental determination using a closed vessel microwave assisted digestion (MARS 5, CEM Corporation, UK). Into each vessel 4 mL of HNO3 and 1 mL of H2O2 was added to accurately weighed toenail and hair samples and left to stand for 30 min before sealing the vessels. The microwave heating program was: 100% power (1,200 W), 5 min ramp to 100°C, held for 2 min, ramped for 5 min to 200°C then held for 30 min. The pressure in the system was approximately 200 psi under these conditions. This method resulted in complete sample dissolution. The solutions were transferred with MQ water to PFA vials and evaporated to dryness on a hotplate at 110°C. Samples were reconstituted with 1 mL of 3% v/v HNO3, heated at 50°C for 10 min and then made up to 3 mL with deionised water to give a final solution of 1% HNO3 for direct determination via ICP-MS.

The enamel and coronal dentine components of teeth were density separated using a heavy liquid method. This was achieved by lightly crushing the decoronated tooth material and adding the powder to the heavy liquid bromoform (CHBr3) in a separating funnel. The bromoform was then slowly diluted with acetone to achieve the optimal density for enamel and dentine separation (2.7 g/mL). The enamel formed sediment at the base of the funnel and was removed. The two components were washed with acetone and dried in a laminar flow hood prior to dissolution. The teeth samples were accurately weighed into acid washed polypropylene autosampler tubes (Sarstedt), to which 0.2 mL of 2:1 HNO3:HCl was added, allowed to dissolve over 5 min, then 0.8 mL of deionised water added, left to stand for 10 min and then made up to a final volume of 10 mL with deionised water. The sample solutions were diluted approximately ×4,000 prior to analysis to ensure a final calcium concentration of 100–200 mg L^−1^, to avoid matrix interference and clogging of the ICP cones and torch. The final matrix prior to analysis contained 1 HNO_3_ and 0.5% HCl. Multielement analysis of toenail, hair and teeth digests was performed by inductively coupled plasma mass spectrometry (ICP-MS, Agilent 7500, Agilent Technologies, UK). The instrument was fitted with a micro flow concentric nebuliser and quartz Scott-type spray chamber. The instrument response was optimised daily using a commercially available Tune solution (SpexCertiprep). Multielement analysis was performed in collision cell mode using He (4 L/min) to minimise potential interferences such as that of the polyatomic ion ^40^Ar + ^35^Cl on ^75^As. An internal standard comprising of Sc, Ge, Rh, In, Te, and Ir was added to the sample line via a T-piece to monitor instrument signal stability. The limit of detection (LOD) for the method expressed as the mean blank signal + 3SD was as follows: Al and Fe < 2 mg/kg; V, As, Se, Mo, Cd < 0.01 mg/kg; Mn and Zn < 0.2 mg/kg; Co, Th and U < 0.005 mg/kg; Cr, Ni, Cu, W, and Pb were <0.07, <0.02, <0.1, <0.08, <0.03 mg/kg respectively. Recoveries for both reference materials were generally better than 100 ± 15% for when compared to available reference values. SPSS version 19 was used for all statistical analyses; an independent sample *T* Test was used to compare metals between two groups. A paired sample two tailed *T* Test was used to compare birth defects in Al Basrah data. Significance level was set at α = 0.05.

## Results and Discussion

It is well-known that exposure to stressors alters the in utero development of a human fetus and has adverse health consequences for the offspring, including a short gestation period, reduced birth weight, increased risk of metabolic, cardiac and psychiatric disease, and overall reduced lifespan (Seckl 1998; Landrigan et al. 2004; Perera et al. 2004; Llop et al. 2010). Populations caught in war-zones or forced to live with severe nutritional restrictions (such as those imposed on the Iraqi population by U.N. sanctions from 1991 to 2003) suffer immediate and chronic stress that leads to long-lasting physical and mental damage. In addition to the harsh effects of sanctions, many Iraqi cities have experienced large-scale bombardment. An accurate tally of the types and volume of ammunition dropped on the Iraqi population is not available. However, reports have indicated that large numbers of bullets have been expended into the Iraqi environment (Buncombe 2011). Thus the environmental contamination of Iraqi cities with materials contained in bullets and bombs may be expected. Toxic metals such as mercury (Hg) and Pb are an integral part of war ammunition and are extensively used in the making of bullets and bombs (Departments of the Army, the Navy, the Air Force, Joint Technical Bulletin 1998; US Department of the Army Technical Manual 1990).

**Fig. 2:**
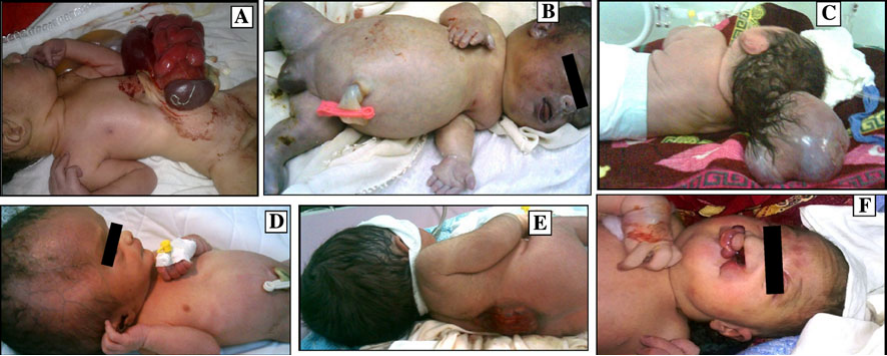
Photos of some birth defects reported from Fallujah General Hospital between May and August 2010. **a** Gastroschisis, **b** Hydrocephalus, **c** Encephalocele, **d** Macrocephaly, **e** Spina Bifida; **f** Cleft lip and palate

**Fig. 3:**
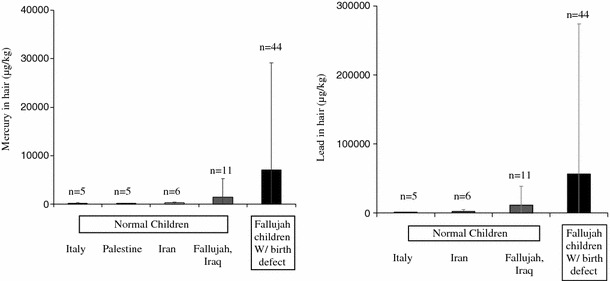
Mercury and lead, (μg/kg, mean ± SD) in hair samples from Fallujah children compared to children from Italy, Palestine, and Iran

**Fig. 4:**
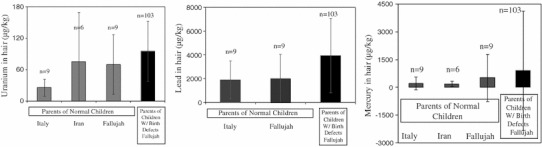
Uranium, mercury and lead, (μg/kg, mean ± SD) in hair samples from parents from Italy, Iran, and Fallujah Iraq

The case study of 56 Fallujah families and the metal analysis of hair samples from this population indicated public contamination with two well-known neurotoxic metals, Pb and Hg. Hair metal data from Fallujah showed Pb to be five times higher in the hair samples of children with birth defects (n = 44; mean ± SD 56,434 ± 217,705 μg/kg) than in the hair of normal children (n = 11; 11,277 ± 27,781 μg/kg). Mercury was six times higher (n = 44; 8,282 ± 25,844 μg/kg Vs n = 11; 1,414 ± 3,853 μg/kg) (Fig. 3). Fallujah mothers who participated in this study did not take any medication and described their diet as “good” during pregnancy. Only one couple was first cousins. Mothers did not drink or smoke during pregnancy. All families consumed water from local aqueducts or locally bottled waters. Siblings of the parents had no history of children with congenital defects. Figure 1 shows a chronological increase in the percentages of birth defects and miscarriages in these Fallujah families. Six photographs of Fallujah children and their conditions are provided in Fig. 2. Mercury and Pb, two toxic metals readily used in the manufacture of present-day bullets and other ammunition, were 6 and 5 times higher in hair samples from Fallujah children with birth defects compared to Fallujah children who appeared normal (Fig. 3). Uranium, Hg and Pb, (μg/kg, mean ± SD) in the hair samples of parents from Italy, Iran, and Fallujah (Iraq), are shown in Fig. 4. Though statistically not significant, the hair of parents of children with birth defects had more uranium, Pb and Hg than the hair of parents of normal children.

The most common abnormalities in Fallujah children were congenital heart defects (n = 24 out of 46), neural tube defects (n = 18 out of 46), and cleft lip/palate (n = 4 out of 46). Cardiac defects, neural tube defects, and facial clefting are known as folate-dependent birth defects since folate intake reduces their occurrence (MRC Vitamin Study Research Group 1991; Wilson et al. 2003; Obican et al. 2010). The Fallujah study has highlighted the role of metals in the manifestation of the current birth defect epidemic in that city. Recent data has linked metal exposure to oxidative stress and folate deficiency in humans (Wang et al. 2012). We also know that in utero metal exposure can culminate in birth deformities by increasing oxidative stress in the womb as the fetus grows (Apostoli and Catalani 2011).

In general, reports of health problems in the Iraqi population and in the surrounding countries have continued to surface (Rajab et al. 2000). News of increases in childhood cancers, of perinatal and infant morbidity and mortality, and of unusual increases in congenital birth defects, have continued to emerge from across Iraq. Data from a central Iraqi city, Al-Ramadi, have corroborated the Fallujah findings (Al-Ani et al. 2010).

Another Iraqi city where birth defects and cancers continue to climb is Al Basrah.

The earliest data on the occurrence of congenital birth defects in Al Basrah came from an article entitled “Incidence of Congenital Fetal Anomaly in Al Basrah Maternity Hospital” (Alsabbak et al. 1997). This research reported on the total number of live births (10,015) in Al Basrah Maternity Hospital between October 1994 and October 1995. The number of birth defects per 1,000 live births during this period was 1.37. Table 1 contains the yearly account of the number of birth defects per 1,000 live births in Al Basrah Maternity Hospital from 2003 to 2011. Central nervous system related defects occurred most frequently. Statistical analysis of this data has shown no significant difference between the number of children born with anencephaly and the number born with Spina Bifida (*p* = 0.28). There were significantly more cases of anencephaly than of hydrocephalus, limb deformity, omphalocele, or short extremities (*p* = 0.009, *p* = 0.005, *p* = 0.000, *p* = 0.000). In addition, the number of Spina Bifida cases was significantly higher than the number of hydrocephalus, limb deformity, omphalocele, or short extremity cases (*p* = 0.05, *p* = 0.005, *p* = 0.000, *p* = 0.001). Within 8 years, the occurrence of congenital birth defects in Al Basrah Maternity Hospital increased by an astonishing 17-fold.

The prevalence of congenital hydrocephalus in California (US) has been reported as 0.6 per 1,000 (Jeng et al. 2011). Worldwide hydrocephalus affects about one in every 1,000 live births. The reported numbers of hydrocephalus from Al Basrah Maternity Hospital were 3.5 times higher than the world average and six times higher than in the United States. Defects of the abdominal wall, like omphalocele and gastroschisis, were also frequently reported in Al Basrah. Omphalocele generally occurs in 0.25/1,000 live births and is associated with a high rate of mortality and severe malformation, such as cardiac anomalies and neural tube defects. The average number of omphalocele observed in Al Basrah Maternity Hospital between 2003 and 2011 was 3.3/1,000 live births.

Neural tube defects (NTDs) occur very early in human development. The prevalence of NTDs in the mainland United States is 1/1,000 live births (CDC; Williams et al. 2002). Some of the highest numbers of NTDs have been reported from coal mining regions in China (10/1,000) (Li et al. 2006). The occurrence of NTDs in Al Basrah (12/1,000) is the highest ever reported and it is increasing. Our data has shown that in Al Basrah, the total number of birth defects more than doubled between 2003 and 2009.

A comparison between the metal levels in the hair (n = 6) and toenail (n = 7) of parents of children with birth defects from Al Basrah, and the associated *p* values, has been presented in Table 2. For most metals (Al, Mn, Co, Cu, Zn, Mo, Pb, Th, and U), hair contained significantly higher amounts of the metal than did toenail, suggesting that hair is a better biomarker of exposure. Examining the Pb hair levels of parents from Al Basrah and Fallujah revealed that the hair of parents of children with birth defects in Al Basrah had 6,500 ± 8,589 (μg/kg); in Fallujah this value was 3,950 ± 3,133 μg/kg; both values being considerably higher than Pb found in the hair samples of parents of normal children from Fallujah (2,012 ± 2,052 μg/kg). The 1.6-fold higher Pb in the Al Basrah parents’ hair compared to Fallujah parents’ hair may be explained by the fact that Al Basrah is an oil-industry dominated area whereas Fallujah is not. Overall, parents of children with birth defects from Al Basrah and Fallujah had twofold, and one-fold, higher Pb in their hair than did parents of normal children respectively. Al Basrah parents who had children with birth defects also had 1.4 times higher enamel Pb (n = 5, 2,497 ± 1,400) than did parents of normal children (n = 5, 1,826 ± 1,619). Additional samples of teeth from the parents of children with and without birth defects from Al Basrah are necessary to help draw reliable statistical conclusions for this population.

**Table 2:** Comparison of metal levels in hair verses toenail from parents of children with birth defects from Al Basrah, Iraq

Metal (mean ± SD) μg/kg	Hair (n = 6)	Toenail (n = 7)	*p* value
Al	84,409 ± 90,923	64,964 ± 34,247	0.06
V	219 ± 185	130 ± 91	0.3
Cr	1,087 ± 869	1,299 ± 410	0.3
Mn	2,848 ± 2,148	1,040 ± 524	<0.0001
Fe	66,318 ± 63,239	63,536 ± 29,029	0.2
Co	78 ± 57	43 ± 17	0.002
Ni	2,489 ± 2835	2,032 ± 2,917	0.8
Cu	16,012 ± 19,593	3,859 ± 870	0.029
Zn	269,486 ± 188,256	116,409 ± 2,1248	<0.0001
As	70 ± 76	102 ± 73	0.9
Se	398 ± 266	659 ± 158	0.5
Mo	143 ± 104	54 ± 19	0.04
Cd	336 ± 554	324 ± 607	0.9
Pb	6,499 ± 8,589	598 ± 107	0.026
Th	22 ± 21	11 ± 5	0.015
U	128 ± 130	12 ± 6	<0.0001

Values are reported as (mean ± SD)

Table 3 contains a literature review of the metal levels in whole deciduous teeth of children from different geographical locations. Enamel is a hard and dense material which is formed during fetal life and it receives small amounts of systemic blood flow thereafter. For this reason it is considered to primarily reflect prenatal exposure to metals. Whole-tooth metal analysis would include enamel, dentin, cementum, and dental pulp. The impact of living in a large city with dangerous levels of air pollution is evident in the high levels of Pb in teeth from Mexico City and Karachi. Similarly, the level of Pb in teeth from Canadian mining areas is indicative of an exposed or impacted population. Mean whole tooth Pb reported from other locations was 1.5 μg/g. Hence, the tooth Pb level of an unimpacted population is more accurately estimated by this 1.5 μg/g value. The enamel portion of the deciduous tooth from a child with birth defects from Al Basrah (4.19 μg/g) had nearly three times higher Pb than the calculated value for a whole tooth from an unimpacted population. Additional samples of deciduous teeth from Al Basrah children with birth defects are necessary to help draw reliable statistical conclusions for this population. Samples of deciduous teeth are currently being collected from impacted (Al Basrah, Iraq) and unimpacted (Isfahan, Iran) areas to enable us to draw meaningful conclusions in this regard. Interestingly, mining, smelting, and living near industry or hazardous waste sites have all been associated with an increased risk of birth defects (Ahern et al. 2011; Zheng et al. 2012; Suarez et al. 2007).

**Table 3:** A literature review of selected metal levels in the whole deciduous teeth of children from various geographical locations verses the sample from Al Basrah, Iraq

Reference and year of the study	Location	Mean lead (μg/g) in deciduous whole tooth of normal children
Barton (2011)	Krakow, Poland	1.6
Tvinnereim et al. (2011)	Addis Ababa, Ethiopia Urban	1
Tvinnereim et al. (2011)	Addis Ababa, Ethiopia Rural	0.33
Rahman and Yousuf (2002)	Karachi, Pakistan	6.4
Hernández-Guerrero et al. (2004)	Mexico City, Mexico	9.1
Tvinnereim et al. (1997)	Norway, 19 counties	1.6
Karahalil et al. (2007)	Ankara and Balikesir, Turkey	1.5
Arruda-Neto et al. (2009)	Brasilia, Brazil	1.3
Bayo et al. (2001)	Cartagena, Spain	3.3
Abdullah et al. (2012)	National, US	0.38
Tsuji et al. (2001)	Ontario, Canada (near smelters)	9.2
Priyanka Prasad PhD Thesis (2010)	Hyderabad and Secunderabad, India	2.26

Present knowledge on the effects of prenatal exposure to metals, combined with our results, suggests that the bombardment of Al Basrah and Fallujah may have exacerbated public exposure to metals, possibly culminating in the current epidemic of birth defects. Large-scale epidemiological studies are necessary to identify at-risk populations in Iraq. The recognition that birth defects reported from Iraq are mainly folate-dependent offers possible treatment options to protect at-risk populations.
